# Brain tumour microstructure is associated with post-surgical cognition

**DOI:** 10.1038/s41598-024-55130-5

**Published:** 2024-03-07

**Authors:** Maite Aznarez-Sanado, Rafael Romero-Garcia, Chao Li, Rob C. Morris, Stephen J. Price, Thomas Manly, Thomas Santarius, Yaara Erez, Michael G. Hart, John Suckling

**Affiliations:** 1 School of Education and Psychology, University of Navarra, 31009 Pamplona, Spain; 2 Department of Medical Physiology and Biophysics, Instituto de Biomedicina de Sevilla (IBiS), HUVR/CSIC/Universidad de Sevilla/CIBERSAM, ISCIII, 41013 Sevilla, Spain; 3 Department of Psychiatry, University of Cambridge, Herchel Smith Bldg, Robinson Way, Cambridge, CB2 0SZ UK; 4 Cambridge Brain Tumour Imaging Laboratory, Division of Neurosurgery, Department of Clinical Neurosciences, University of Cambridge, Cambridge, CB2 0QQ UK; 5 Department of Applied Mathematics and Theoretical Physics, The Centre for Mathematical Imaging in Healthcare, Cambridge, CB3 0WA UK; 6 Academic Neurosurgery Division, Department of Clinical Neurosciences, University of Cambridge, Cambridge, CB2 0QQ UK; 7 MRC Cognition and Brain Sciences Unit, University of Cambridge, Cambridge, CB2 7EF UK; 8 Faculty of Engineering, Bar-Ilan University, 5290002 Ramat Gan, Israel; 9 St George’s, University of London and St George’s University Hospitals NHS Foundation Trust, Institute of Molecular and Clinical Sciences, Neurosciences Research Centre, Cranmer Terrace, London, SW17 0RE UK; 10 Cambridge and Peterborough NHS Foundation Trust, Cambridge, CB21 5EF UK; 11 School of Medicine & School of Science and Engineering, University of Dundee, Dundee, DD1 4HN UK; 12 The Gonda Multidisciplinary Brain Research Center, Bar-Ilan University, Ramat Gan, Israel

**Keywords:** Brain tumors, Tumour microstructure, Microstructure, Diffusion MRI, Neurosurgery, Cognitive neuroscience, Diseases of the nervous system, Surgical oncology, Cancer imaging

## Abstract

Brain tumour microstructure is potentially predictive of changes following treatment to cognitive functions subserved by the functional networks in which they are embedded. To test this hypothesis, intra-tumoural microstructure was quantified from diffusion-weighted MRI to identify which tumour subregions (if any) had a greater impact on participants’ cognitive recovery after surgical resection. Additionally, we studied the role of tumour microstructure in the functional interaction between the tumour and the rest of the brain. Sixteen patients (22–56 years, 7 females) with brain tumours located in or near speech-eloquent areas of the brain were included in the analyses. Two different approaches were adopted for tumour segmentation from a multishell diffusion MRI acquisition: the first used a two-dimensional four group partition of feature space, whilst the second used data-driven clustering with Gaussian mixture modelling. For each approach, we assessed the capability of tumour microstructure to predict participants’ cognitive outcomes after surgery and the strength of association between the BOLD signal of individual tumour subregions and the global BOLD signal. With both methodologies, the volumes of partially overlapped subregions within the tumour significantly predicted cognitive decline in verbal skills after surgery. We also found that these particular subregions were among those that showed greater functional interaction with the unaffected cortex. Our results indicate that tumour microstructure measured by MRI multishell diffusion is associated with cognitive recovery after surgery.

## Introduction

Malignant brain tumours are a particularly lethal form of cancer due partly to the close entwinement of parenchymal tissue and precursor cells that is critical for their instantiation and subsequent progression. The effect of tumours on the functional brain networks in which they become embedded remains enigmatic. A better understanding of the effects of tumours on cognition, and perhaps more importantly on onco-functional balance, are crucial for planning of disease management, including surgery and radio- and chemo-therapy.

It is well known that brain tumours are heterogeneous in microstructure and vasculature not only between individuals, but also within an individual tumour. Microstructural imaging techniques give information about tissue features on the cellular level without relying on invasive procedures^1^. In particular, magnetic resonance imaging (MRI) can provide insightful in vivo information on cancer biology relevant to disease diagnosis, prognosis, and response to treatment^2^. Regional microstructural features extracted from MRI have been found to be related to clinical outcomes.

Within the published literature, intra-tumoural microstructural patterns have been shown to predict survival of glioblastoma patients with approaches for determining tumour microstructure quantified by histogram-based measures, shape and volume-based measures and texture analysis^3^. Specifically, a higher proportion of decreased isotropic diffusion and increased anisotropic diffusion regions within the tumour obtained from 2D histogram-based analyses has been associated with lower rates of survival^4^. Similarly, spatial features obtained from specific tumoural subregions are predictive of survival times and survival rates^5,6^. In these approaches, spatial features were either obtained from contrast-enhanced T1-weighted, FLAIR, T2-w images, or diffusion weighted MRI. Additionally, texture analyses based on MRI discriminates between gliomas and metastasis^7,8^, between high- and low-grade gliomas^9^, and between oedema, tumoural and normal brain areas^10^.

Among the variety of MR imaging techniques, diffusion imaging is capable of assessing changes in brain microstructural anisotropy from water diffusion by applying several directional gradients during acquisition. For instance, the relationship between apparent diffusion coefficient (ADC) and cellularity can be used to detect dense tumours and even probe the efficacy of treatment over time^1^. In addition, diffusion tensor imaging (DTI) can predict tumour infiltration and potential invasive migration of malignant cells^2,11^. The neurite orientation dispersion and density imaging (NODDI) technique uses multiple varying diffusion gradient strengths to provide more specific measurements of neurite morphology, such as their density and orientation dispersion^12–14^^.^

Our previous work indicates that the degree of functional coupling between the residual tumour (i.e., after surgical resection) and healthy functional brain networks is related to cognitive recovery at 12 months follow-up^15^. Specifically, patients that showed a greater reduction in glioma–global signal coupling after surgery were more likely to suffer greater cognitive difficulties in the long-term. High preoperative neurite density in the margins of the tumour, and also within the default mode network, has also been associated with better memory recovery^16^. Together, these findings indicate that tumours may play an active role in brain function, and thus their resection has a direct bearing on cognitive outcomes. However, the role of tumours on the functional brain networks in which they become embedded remains enigmatic.

The rationale of this study is to integrate structural and functional pre-operative imaging of brain tumours to investigate the hypothesis that tumour microstructure impacts cognitive recovery following surgery through the functional embedding of tumours within brain functional networks. To test this hypothesis, we first characterized tumour microstructure by dividing the tumour into different subregions. To assess the robustness of the results, we implemented two different methodological approaches. The first used DTI p- and q-images and a two-dimensional histogram-based method for image segmentation^17^ that has previously been successful in partitioning and quantifying heterogenous tumours^4,18,19^. The second was a data-driven approach for intra-tumour segmentation using information from NODDI images in a two-dimensional feature space. Specifically, we used a Gaussian mixture model^20^ to characterize feature space density^21–23^.

Once the tumour subregions were identified in the pre-operative state, we evaluated which had a greater impact on participants’ cognitive recovery after surgery. Finally, we examined the role of tumour microstructure in the functional interactions between the tumour and the unaffected brain in the pre-operative state. Tumour subregions demonstrating the highest functional coupling with the unaffected brain were expected to play a leading role in cognitive recovery after surgery.

The results of the present work are designed to extend our understanding of the factors influencing cognitive outcomes for patients with brain tumours that could positively impact the onco-functional balance of treatment.

## Materials and methods

### Methodological strategy

**Figure 1:**
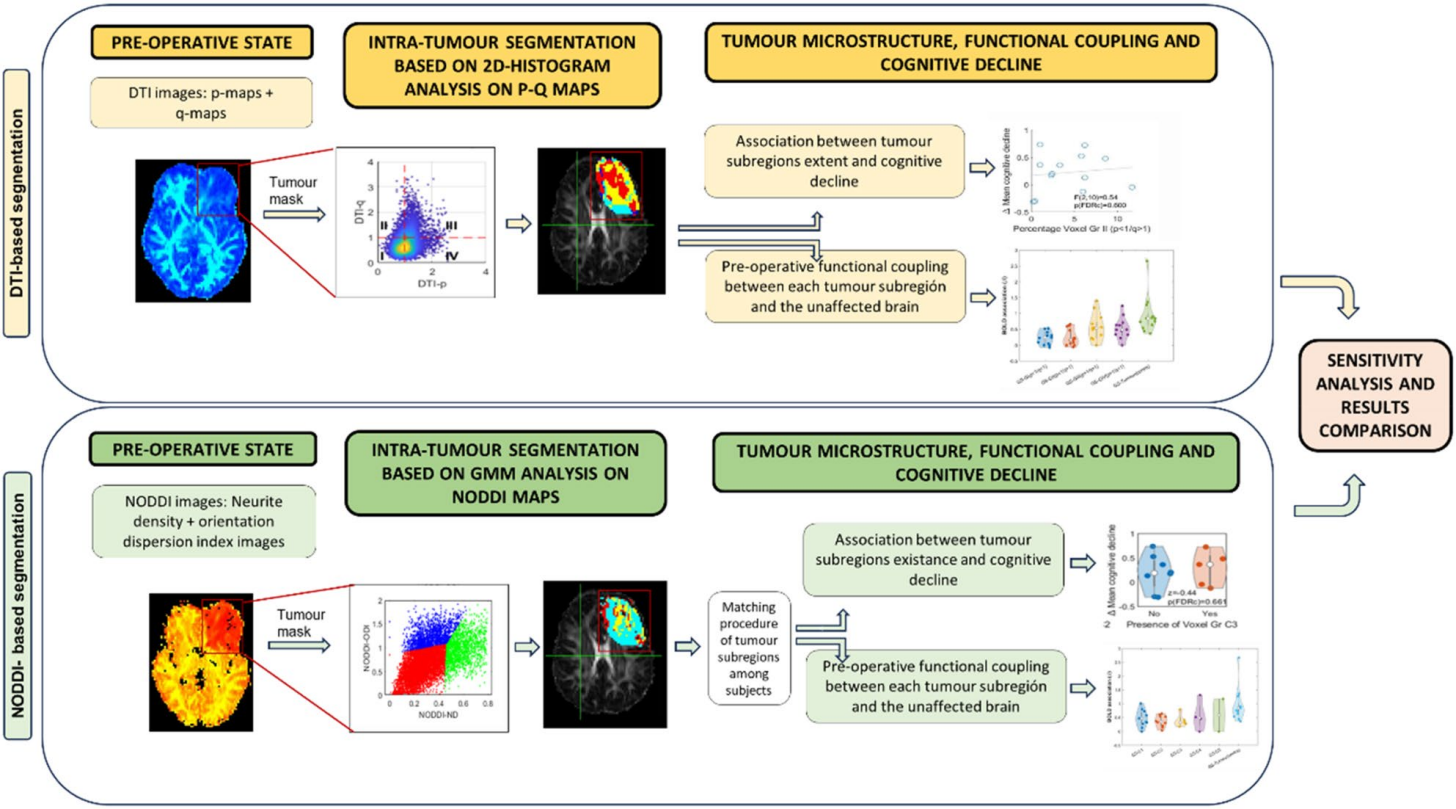
Flowchart of data processing and analysis. Two methodological approaches (DTI -top- and NODDI -bottom-) were used to identify tumour subregions according to its microstructure. Segmented tumours were used to separately analyse the correlation between BOLD signal within each tumour subregion and the Global Signal from the healthy brain. Additionally, the presence of tumour subregions was also correlated with cognitive decline after surgery.

Methodological steps are summarised in Fig. 1. First of all, tumour subregions were identified by analysing the intra-tumoral microstructure, quantified from diffusion-weighted pre-operative MRI scans. We initially pre-processed MRI images and masked the tumour regions (see details in Sects.  “MRI data acquisition and pre-processing” and “Tumour masking and image co-registration”). Two different strategies were used for characterizing tumour microstructure based on: (i) histogram analyses of DTI images (P and Q maps; see Sect.  “Segmentation from DTI”) and: (ii) general mixture modelling of NODDI neurite density and NODDI orientation dispersion index images (see Sect.  “Segmentation from NODDI”). Subsequently, tumour subregions that had a greater impact on participants' cognitive recovery after surgery were identified. To reduce the impact of the variability across participants prior to the intervention, cognitive recovery was calculated as the change in cognitive function from the pre-operative to the post-operative period. Three participants lacked neuropsychological assessments in the post-operative period and therefore were not included in these analyses. These analyses are described in Sect.  “Associations of cognitive change with DTI-identified tumour subregions” (DTI approach) and in Sect.  “Associations of cognitive change with NODDI-identified tumour subregions” (NODDI approach). The similarity level between the identified tumour subregions using both methodological approaches was subsequently assessed.

Finally, we studied the role of tumour microstructure in the functional interaction with the unaffected brain during the pre-operative state. We expected that tumour subregions demonstrating the highest functional coupling with the unaffected brain in the pre-operative state would play a key role in cognitive recovery after surgery.

### Sample

This prospective cohort and all experiments were approved by the Cambridge Central Research Ethics Committee (Reference number 16/EE/0151), according with relevant guidelines and regulations. All patients gave written informed consent before participating in the study.

Those patients with a typical appearance of a diffuse glioma were identified at adult neuro-oncology multidisciplinary team (MDT) meetings at Addenbrooke's Hospital (Cambridge, UK). A consultant neurosurgeon directly involved in the study identified potential participants based on the outcome of the MDT discussion. Eighteen patients aged 22–56 years (seven females) were finally approached to take part in the study. Inclusion criteria were: (i) participant was willing and was able to give informed consent; (ii) imaging was evaluated and judged to have typical appearances of a diffuse non-enhancing glioma; (iii) Stealth MRI was obtained (a routine neuronavigation MRI scan performed prior to surgery); (iv) World Health Organisation (WHO) performance status was 0 or 1; (v) age was between 18 and 80 years; (vi) tumour was located in or near speech-eloquent areas of the brain, i.e., regions that might be critical for speech comprehension and articulation; and (vii) patients could undergo awake surgical resection of a diffuse glioma. Participants were excluded if any of the following applied: (i) concomitant anti-cancer therapy; (ii) history of previous malignancy (except for adequately treated basal and squamous cell carcinoma or carcinoma in situ of the skin) within 5 years; and, (iii) previous severe head injury. Contrast enhancement and oedema (defined as vasogenic oedema / hypointensity on FLAIR) was excluded enrolment.

One participant withdrew due to not being able to tolerate the MRI environment. Since one of the main objectives of the present work was to measure intra-tumoural microstructure , data from a participant with a recurrent tumour was also excluded. Thus, data from sixteen participants were included in the analyses (see Table 1 for demographics, tumour/treatment information and histological assessment of the tumours).

**Table 1:** Demographic and pathological information of participants included in the study. # Number of participant; M: Male; F: Female; L: Left; R: Right; SFG: Superior Frontal Gyrus; MFG: Middle Frontal Gyrus; IFG: Inferior Frontal Gyrus; ITG: Inferior Temporal Gyrus; MTG: Middle Temporal Gyrus; SMA: Supplementary Motor Area; RT: radiotherapy; Astro: astrocytoma; GBM: glioblastoma multiforme; Oligo: oligodendroglioma; Lesion Vol Pre/Post: Total volume occupied by the tumour (Pre) and total amount of damaged tissue (Post) according to the lesion mask manually drawn on the MPRAGE image and refined with Unified Segmentation Lesion toolbox.

#	Age	Sex	Years Educa- Tion	Hand-edness	Presen-tation	Hemi	Location	Histology (WHO grade)	Molecular signature	Tumour/ Lesion Vol (Pre/Post) (cm3)	**Treat-ment**
1	41	F	18.5	L	Seizures	L	Frontal	Oligo (II)	*IDH* Mutated *1p19q* Lost *ATRX Retained*	166.8 94.5	Chemo-RT
2	32	M	17.2	R	Seizures	R	Insula	Astro (II)	*IDH WT* *1p19q -* *ATRX Lost*	83.2 47.8	Chemo-RT
3	26	M	11.5	R	Seizures	L	Temporal / Insula	GBM (IV)	*IDH* Mutated *1p19q -* *ATRX Lost*	59.0 30.1	Chemo-RT
4	49	F	29.5	R	Incidental	R	Insula	Oligo (II)	*IDH* Mutated *1p19q* Lost *ATRX Retained*	31.5 37.5	Observation
5	22	F	16	L	Seizures	R	Frontal / IFG	Ganglio-glioma (I)	*IDH -* *1p19q -* *ATRX -*	4.4 7.5	Observation
6	29	M	11.5	R	Seizures	R	Frontal / SFG & MFG	Astro (III)	*IDH* Mutated *1p19q* Negative *ATRX Lost*	31.8 70.0	Chemo-RT
7	29	M	12	R	Seizures	R	Frontal / MFG	Astro (III)	*IDH* Mutated *1p19q* Negative *ATRX Lost*	22.6 34.2	Chemo-RT
8	50	M	10	L	Seizures	L	Temporal / ITG	GBM (IV)	*IDH WT* *1p19q* Negative *ATRX Retained*	15.6 15.7	Chemo-RT
9	38	F	16	R	Seizures	L	Frontal / MFG	Oligo (II)	*IDH* Mutated *1p19q* Lost *ATRX Retained*	34.7 54.9	Chemo-RT
10	29	M	10	R	Seizures	L	Frontal / SFG / frontal pole	Astro (II)	*IDH* Mutated *1p19q* Negative *ATRX Lost*	52.1 116.9	Observation
11	33	F	14	R	Head-aches	L	Temporal / MTG	Astro (III)	*IDH* Mutated *1p19q* Negative *ATRX Lost*	87,0 84,8	Chemo-RT
12	27	F	13	R	Seizures	L	Superior Temporal Gyrus	Ganglioglioma (I)	*IDH WT* *1p19q -* *ATRX -*	7.6 8.5	Observation
13	56	F	14.5	R	Seizures	L	Superior Temporal Gyrus	Astro (II)	*IDH* Mutated *1p19q* Negative *ATRX Lost*	37.4 19.2	Chemo-RT
14	32	M	11	R	Seizures	L	Superior Temporal Gyrus	Astro (III)	*IDH Mutated* *1p19q -* *ATRX Lost*	18.0 30.3	Chemo-RT
15	27	M	16	R	Seizures	L	SFG/SMA & Pre-central	GBM (IV)	*IDH WT* *1p19q Negative* *ATRX Retained*	73.6 43.7	Chemo-RT
16	30	M	14	R	Seizures	L	Inferior frontal	Astro (III)	*IDH Mutated* *1p19q -* *ATRX Lost*	26.2 17.4	Chemo-RT

Each participant was scanned up to four times: before surgery (pre-operative), within 72 h after surgery, and at 3 and 12 months after surgery. Only pre-operative scans were analysed in this study. The fMRI images from these participants were previously analysed^15,24^.

### Neuropsychological assessment

Participants were given a neuropsychological assessment two weeks before surgery (pre-operative) and between two and five weeks after surgery (post-operative). In two cases, post-operative neuropsychological assessment was performed around six months after surgery (see Table S2). Testing was administered by a neuropsychologist in a clinical setting and took approximately 2–3 h to be completed (more information about the tests can be found in SI). The cognitive domains assessed in this study were: memory (verbal and nonverbal), verbal skills, nonverbal skills, attention, and executive function. Item-level details can be found in Table S1.

### MRI data acquisition and pre-processing

Participants were scanned at the Wolfson Brain Imaging Centre, University of Cambridge using a Siemens Magnetom Prisma-fit 3 Tesla MRI scanner and a 16-channel receive-only head coil (Siemens AG).

Anatomical MRIs were acquired using a T1-weighted MPRAGE sequence: repetition time (TR) = 2300 ms, echo time (TE) = 2.98 ms, flip angle (FA) = 9°, 1 mm^3^ resolution, field of view (FOV) = 256 × 240 mm^2^, 176 contiguous slices.

Multishell diffusion sensitive data were acquired with the following parameters: TR = 8200 ms, TE = 95 ms, 2.5mm^3^ resolution, 60 slices, FOV = 240 × 240 mm^2^, acquisition of 30 directions with b-value = 800 mm/sec, 60 directions with b-value = 2000 mm/sec, and 10 unweighted B0 images.

DTI maps were obtained using the diffusion toolbox of FSL (fsl.ox.ac.uk). Corrections for B0 field inhomogeneity, Gibbs artifacts, and eddy-current distortions were applied using MRtrix v3 (https://www.mrtrix.org/).

The NODDI Matlab Toolbox (mig.cs.ucl.ac.uk/index.php?n = Tutorial.NODDImatlab) used the diffusion imaging data for quantification of in vivo microstructural complexity of dendrites and axons^12^. The NODDI multi-compartment tissue model extracts two key contributing factors to fractional anisotropy: the Gaussian contribution from water molecules located in the extracellular space, and the restricted non-Gaussian diffusion that takes place in the intracellular space. The apparent intracellular volume fraction was used in this work as a measure of neurite density (ND). The orientation dispersion index (ODI), a measure of the orientation coherence of neurites, was also calculated.

Resting-state (eyes closed) fMRI was acquired with a BOLD-sensitive sequence: TR = 1060 ms, TE = 30 ms, acceleration factor = 4, FA = 74°, 2 mm^3^ resolution, FOV = 192 × 192 mm^2^. Pre-processing of fMRI images included slice timing correction, bias field correction, rigid body motion correction, normalisation by a single scaling factor and smoothing to 5 mm full-width half-maximum. Independent component analysis (ICA) was performed with FSL MELODIC after which noise components were identified and removed using ICA-FIX^25^ with training specific to this dataset^26^. Wavelet filtering was used to retain the BOLD oscillations in the physiologically relevant frequency range 0.03–0.12 Hz^27^.

### Tumour masking and image co-registration

Probabilistic masks of each pre-operative tumour were generated using a semi-automated procedure and the anatomical MRI (a detailed description can be found in SI). All tumour masks were binarized by applying a threshold of 0.5 to the probabilistic mask.

### Intra-tumour segmentation

We quantified tumour microstructure to capture intra-tumoral characteristics. Two different approaches were carried out independently using the diffusion images: one based on DTI images, and the other based on NODDI neurite density and NODDI orientation dispersion index images. Since only pre-operative scans were used in the analyses and vasogenic oedema was an exclusion criterion, the presence of oedema was limited.

#### Segmentation from DTI

For each participant, DTI-p (isotropic) and DTI-q (anisotropic) components were initially calculated^28^ at each voxel within the tumour mask, and a feature space using the p- and q-values generated. Each value was normalized by dividing it by the mean value in the contralateral tumour region such that values approaching 1 represent diffusion patterns similar to unaffected tissue. The joint histogram of p and q values was created with each quadrant around the [1, 1] origin denoting partitions of the feature space, as previously described^4^ (see Fig. S1A for a flowchart of the analysis):

*Group I:* Decreased DTI-p/decreased DTI-q; p < 1, q < 1.

*Group I*I: Decreased DTI-p/increased DTI-q; p < 1, q > 1.

*Group III*: Increased DTI-p/increased DTI-q; p > 1, q > 1.

*Group IV*: Increased DTI-p/decreased DTI-q; p > 1, q < 1.

Finally, the partition labels of each point in the feature space were translated back to the voxels of the DTI space generating four tumour subregions. A binary mask was created for each tumour subregion.

#### Segmentation from NODDI

Neurite density and orientation dispersion index images derived from the NODDI modelling were used to perform a feature space analysis (see Fig. S1B for a flowchart of the analysis). In the same way as the analysis of DTI, for both ND and ODI images voxels within the tumour were selected by applying the binarized mask and normalised by the mean values in the contralateral tumour region. The feature space of ND and ODI values was then created from the normalised values.

Only 4 participants had values of ND > 1 within the tumoural region. Therefore, a quadrants division around the [1, 1] origin to define tumour partitions was inappropriate. Instead, an unsupervised data-driven approach for intra-tumour partitioning was adopted. Specifically, a Gaussian mixture modelling (GMM) (covariance type: full, shared covariance: true, 5 replicates) was applied to the joint 2D ND-ODI histogram to generate a data-driven partition of the feature space. The parameters of the model were estimated by the expectation maximization (EM) algorithm. Outliers were removed before applying GMM^29^ using Matlab. The number of partitions in the GMM (range: 1–7) was set to that maximizing the silhouette index. One half of the participants had two partitions with the remainder having three partitions. Unlike the p-q approach which forces a 4-class solution, tumours did not present with the same number of partitions, nor were the partitions in similar locations of the feature space. To identify common partitions across participants, a matching procedure was required to find those partitions among all the tumours that had the minimum Euclidean distance between the centroids of their distributions. To ensure a good match, an additional requirement of Eucledian distance between centroids < 0.3 was imposed to find a match. This step was performed in an iterative manner matching all the partitions across all tumours. No satisfactory match was found for two partitions of two different tumours, and therefore these two partitions were excluded from further analysis. In total, five partitions were identified across tumours, denoted: C1, C2, C3, C4 and C5.

Finally, partition information from the feature space was translated into the NODDI space generating tumour subregions for each participant. A binary mask was created for each tumour subregion.

### Tumour microstructure and patients’ cognition

To mitigate the potential bias arising from relying solely on post-operative cognitive state scores and to account for baseline variations, we opted to address individual differences in cognitive performance by subtracting the post-operative z-score from the pre-operative z-score for each cognitive domain (attention, non-verbal skills, memory, verbal skills and executive function). In this way, the longitudinal trajectory of the assessments (Δ) was tracked, where positive scores represent a deterioration in performance relative to pre-operatively (i.e., cognitive decline). Mean overall cognitive change was calculated for each participant as the average of the z-scores obtained across all cognitive domains.

#### Associations of cognitive change with DTI-identified tumour subregions

Linear regression analyses were carried out to test the association between mean overall cognitive decline and the percentage of occupancy of the four tumour subregions identified within the tumour. Separate analyses were performed for each of the p-q subregions (I, II, III, IV), with the mean overall cognitive decline as the dependent variable and the percentage occupancy of the tumour by the evaluated subregion as the independent variable. Tumour volume was included as a covariate.

If the percentage occupancy of one tumour subregion was a significant predictor of mean overall cognitive decline, we then conducted univariate regression analyses for each cognitive domain. This allowed identification of which cognitive domain had the greatest effect size associated to that specific subregion.

We also investigated whether the percentage occupancy of a specific subregion changed significantly with the presence (or not) of IDH mutation using a Mann Whitney statistical test.

#### Associations of cognitive change with NODDI-identified tumour subregions

As tumours did not have the same number or type of partitions, we determined what effect the presence of a certain subregion had on cognitive decline. Regression analysis including the percentage occupancy of a specific subregion was not appropriate in this case since some of the partitions were only present in a reduced number of tumours (i.e. C5 in 2 tumours and C3 and C4 in 5 tumours). For this reason, the association between cognitive change and tumour microstructure was tested, for each of the subregions (C1, C2, C3, C4 and C5), by comparing the cognitive change in the group of tumours that presented with a certain subregion with the cognitive change of those participants in which it was not present. Non-parametric Mann–Whitney tests were used for these analyses. We also investigated whether the presence of a specific subregion was significantly associated with the presence (or not) of an IDH mutation (Pearson’s chi-squared test). Both in DTI and NODDI approaches, all p-values were corrected for the Benjamini–Hochberg false discovery rate (FDR < 0.05) to reduce the likelihood of false positives.

Given our reduced sample size, we avoided including too many covariates in our models to prevent overfitting. However, variables such as years of education, age, pre- and post-surgical tumour/lesion volume, tumour grade, pre-surgical hippocampal volume or pre-surgical hippocampal activity may be associated with cognitive decline. To assess whether any of these variables were potential predictors of cognitive decline, we calculated the Pearson correlation coefficient between these variables and mean overall cognitive decline scores.

#### Spatial similarity between DTI- and NODDI-identified subregions

To assess the spatial similarity of the NODDI and p-q subregions that were significantly associated with cognitive change, the Sørensen-Dice similarity coefficient and the percentage occupancy (%Occ) were calculated for each NODDI-identified subregion within the specific p-q subregion (and vice versa). %Occ is a metric used to quantify the extent to which one specific subregion occupies or overlaps with another subregion. Its calculation involves determining the percentage of voxels within a particular subregion that coincide with or are shared by another subregion. %Occ was computed by taking the number of overlapping voxels between two specified subregions and expressing it as a percentage of the total voxels in the subregion of interest. This measurement provides insight into the spatial relationship and extension of one subregion in relation to another. This value was calculated as a complementary measure to the DICE coefficient to provide a more comprehensive evaluation of the spatial relationships between two specified subregions.

### Time-series analyses

To understand the relationship between tumour microstructure and functional interactions, the functional coupling between the BOLD time-series of each tumour subregion and the time-series from the rest of the brain was calculated.

First, the binary masks corresponding to each tumour subregion were linearly transformed from DTI to T1-weighted anatomical space and then from this space to fMRI space using advanced normalization tools (ANTs). BOLD signals were then extracted and averaged across voxels for: (1) each of the tumour subregions; (2) the tissue contralateral to the tumour; and, (3) in all grey matter (GM) excluding the whole tumour. The average BOLD signal extracted from healthy GM constituted the global signal (GS).

Time-series analysis was performed separately for DTI- and NODDI-identified tumour subregions. Outlying images based on framewise displacement were removed (see detailed description in SI). The functional coupling between the time-series of the subregions and the GS, and the association between the time-series of the contralateral tumour region and the GS were measured. This association, β, was calculated as the slope relating two BOLD time-series in a linear regression analysis using two different approaches: (i) a per-voxel functional analysis that calculated β between GS and each voxel within the tumour; and (ii) a subregion-wise analysis that averaged timeseries within each tumour subregion, and then calculating β between the GS and the averaged BOLD signal.

Subsequent analyses were performed with the (ii) subregion approach. Non-significant β values (p-FDR corrected > 0.05) were found in eight cases for DTI-derived tumour subregions and in three cases for NODDI-derived tumour subregions. In these cases, β association values were set to 0, since there was no evidence that the β value was significantly different from 0. The β values corresponding to the association between the time-series of the contralateral tumour region and the GS were considered as an average measure of functional coupling between healthy tissue and the GS. In this case, the GS was calculated excluding the voxels of the tumour as well as those of the contralateral tumour region.

Non-parametric Friedmann statistical tests were used to compare β values across the different p-q subregions and contralateral tumour. Post-hoc analyses were performed with non-parametric Wilcoxon signed-rank tests. FDR correction was applied to post-hoc comparisons. A Friedmann comparison test was not carried out using β values of NODDI-derived subregions due to the fact that not all the subregions were present in all the tumours, and because of the low sample size of the subregions C3 (n = 5), C4 (n = 4) and C5 (n = 2).

## Results

### Intra-tumour segmentation

#### Segmentation from DTI

The percentage of each subregion’s occupancy within the tumour (i.e., the percentage that a subregion occupies in relation to the total number of voxels of the tumour) was averaged across participants (see Table 2, DTI approach). Replicating the results of Li et al.^4^, group IV (p > 1, q < 1) occupied the largest proportion of the tumour, followed by group III (*p* > 1, q > 1).

**Table 2:** DTI approach. Mean percentage occupancy of each DTI-derived subregion within the tumour. DTI: diffusion tensor imaging; p: isotropic component; q: anisotropic component; SD: standard deviation. NODDI approach**.** Characteristics of each subregion (C1, C2, C3, C4 and C5) defined from NODDI joint histogram analysis: mean ND and ODI values of each Group; percentage of patients that had each Group within the tumour; mean percentage occupancy of each subregion within the tumour, including only the participants that had that subregion. NODDI: neurite orientation dispersion and density imaging; ND: neurite density; ODI: orientation dispersion index; SD: standard deviation.

DTI approach				
DTI Joint histogram subregions	Mean (%) ± SD			
Group I (*p* < 1, q < 1)	11.2 ± 10.9			
Group II (*p* < 1, q > 1)	4.3 ± 3.3			
Group III (*p* > 1, q > 1)	27.9 ± 11.5			
Group IV (*p* > 1, q < 1)	56.6 ± 10.8			

NODDI approach				
NODDI Joint histogram subregions	Mean ND values (SD)	Mean ODI values (SD)	% patients	Mean (%) ± SD
Group C1	0.34 (0.09)	0.63 (0.09)	87.5	45.0 ± 26.4
Group C2	0.41 (0.18)	1.11 (0.17)	75	22.8 ± 10.6
Group C3	0.56 (0.04)	0.79 (0.12)	31.25	25.2 ± 22.3
Group C4	0.16 (0.03)	0.46 (0.08)	31.25	74.6 ± 7.2
Group C5	0.12 (0.02)	0.28 (0.04)	12.5	73.2 ± 7.2

**Figure 2:**
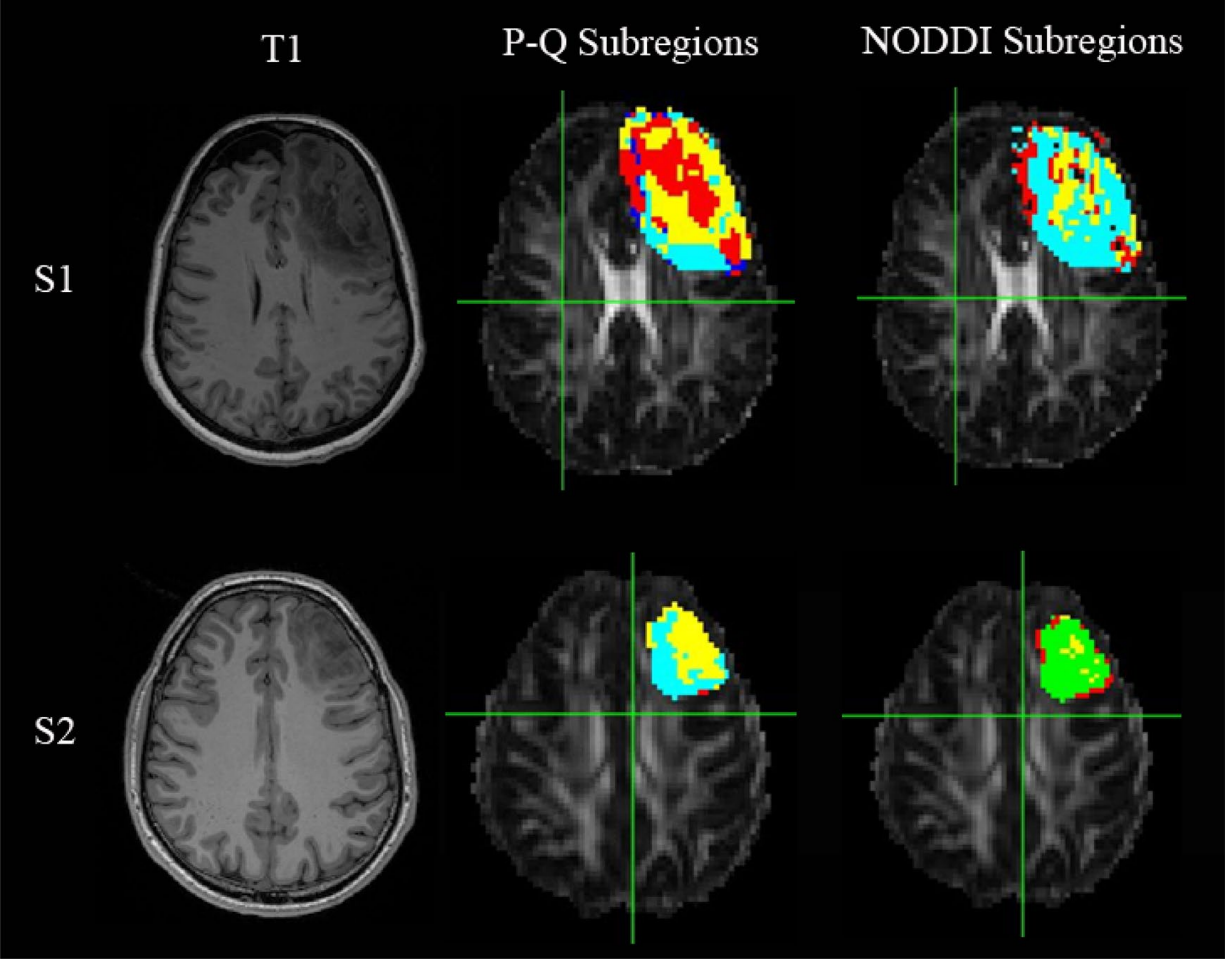
Illustration of the spatial distribution of the different voxel groups within the tumour in two different participants (first row: Participant 1 (S1), second row: Participant 2 (S2)). (Left) T1 weighted image. (Middle) p-q-derived tumour subregions. Voxel group I is displayed in red; Voxel group II: dark blue; Voxel group III: Light blue and Voxel group IV: yellow. (Right) NODDI-derived tumour subregions: C1 is displayed in light blue; C2: yellow; C3: red; C4: green.

An illustration of the spatial distribution of tumour subregions in two different participants is shown in Fig. 2.

#### Segmentation from NODDI

As a result of the data-driven segmentation procedure, not all subregions derived from NODDI modelling were present in all tumours. Table 2 (NODDI approach) presents the percentage of tumours that had specific subregions identified from NODDI modelling: C1, C2, C3, C4 or C5. Less than 13% of the tumours had subregion C5, C3 and C4 were present in 31% of the tumours, and most of the tumours had subregions C1 (87.5%) and C2 (75%). Where present, the mean occupancy of each subregion within the tumour was also calculated. C4 and C5 were associated with the highest occupancy rates.

An illustration of the different spatial distributions of subregions within the tumour in two different participants is displayed in Fig. 2.

### Tumour microstructure and patients’ cognition

#### Associations of cognitive change with DTI

**Figure 3:**
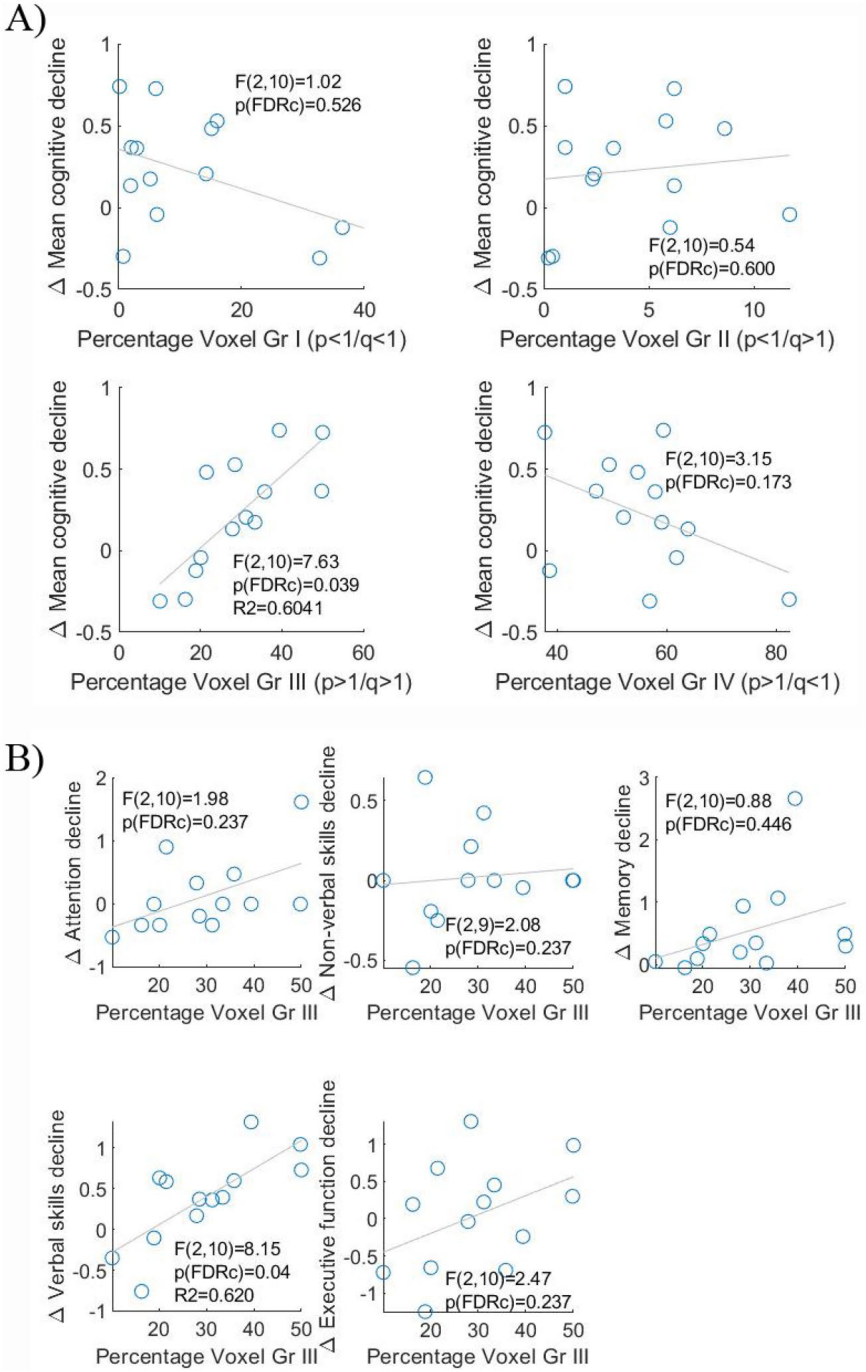
(**A**) Association of mean cognitive decline after surgery with the percentage occupancy of each p-q-derived subregion (groups I, II, III and IV). (**B**) Association of mean cognitive change after surgery and the percentage occupancy of p-q-derived group III subregion for each of the 5 cognitive domains (attention, non-verbal skills, memory, verbal skills and executive function).

To assess the relationship between tumour microstructure and cognition, we evaluated first whether the percentage occupancy of a specific subregion within the tumour was associated with the participant’s mean overall cognitive change after surgery (Fig. 3A). We found that mean overall cognitive change was significantly predicted by the proportion of group III voxels present within the tumoural region.

The results of the regression indicated that the model explained 60% of the variance (R^2^ = 0.6041, F(2,10) = 7.63, p-FDR = 0.039) and that higher proportions of group III within the tumour contributed significantly to greater overall cognitive decline (B = 0.022, p = 0.004). Tumour volume was not a significant predictor (p = 0.970) of overall cognitive change. Therefore, the proportion of group III within the tumour predicted pre- to post-surgical change in cognitive status. The models that included percentage occupancy of groups I, II and IV did not contribute significantly to overall cognitive change (Group I: F(2,10) = 1.02, p-FDR = 0.526; Group II: F(2,10) = 0.54, *p*-FDR = 0.600; Group IV: F(2,10) = 3.15, p-FDR = 0.173).

Since the extent of group III was associated with mean cognitive change, we investigated whether this predictive capability was related to a specific cognitive domain; namely, attention, non-verbal skills, memory, verbal skills or executive function. We found that the proportion of group III within the tumour was a significant predictor of a change in verbal skills. The results of the regression indicated that the model explained 62% of the variance (R^2^ = 0.620, η^2^ = 0.619, F(2,10) = 8.15, p-FDR = 0.04) and that higher proportions of group III within the tumour were associated with greater verbal skills decline (B = 0.037, p = 0.002). Decline in other cognitive domains was not predicted by the percentage occupancy of group III subregion (Fig. 3B). The percentage occupancy of group III did not change significantly with the presence (or not) of IDH mutation (Mann Whitney, z = -1.64, p = 0.100).

#### Associations of cognitive change with NODDI

**Figure 4:**
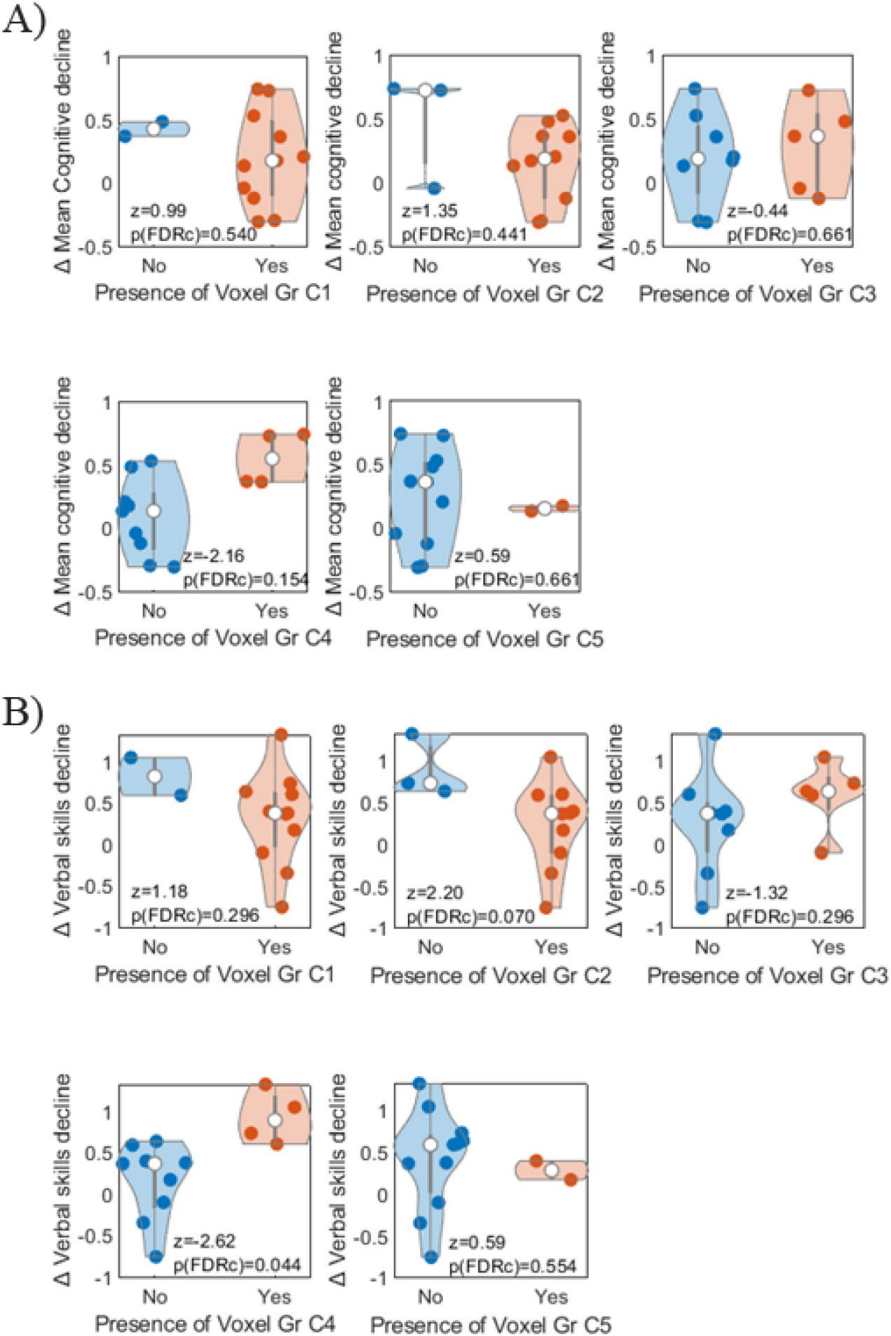
Distribution of decline in mean cognition (**A**) and verbal skills (**B**) across participants (represented by individual dots) depending on the presence or not of each NODDI-derived subregion (C1, C2, C3, C4 and C5).

Since not all subregions derived from NODDI modelling were present in all tumours, we assessed whether cognitive change in each domain was significantly different for patients that did or did not have a specific subregion (Fig. 4A). Mean change in overall cognition was not associated with the presence of any subregion. Only subregion C4 had an uncorrected p-value of 0.03 that did not survive FDR correction.

Focusing on the cognitive domain where the extent of p-q groups had previously shown significant associations, we next assessed whether verbal skills changed significantly in the presence of specific NODDI subregions (Fig. 4B). In this case, participants that had the subregion C4 within the tumour showed a significant decline in verbal skills compared to those participants without that subregion (η^2^ = 0.529, z = −2.62, *p*-FDR = 0.044). Tumour volume did not vary significantly for participants who had or did not have the subregion C4 (z = 0.309, *p* = 0.758).

We also determined that the presence of C4 subregion was not significantly associated with the presence of IDH mutation (Pearson’s chi-squared, χ^2^ = 0.428, p = 0.513).

To assess whether other demographic or clinical characteristics were also potential predictors of cognitive decline after surgery, the Pearson correlation coefficient between these characteristics and mean overall cognitive decline was calculated. No significant correlations were found between potential predictors and mean overall cognitive decline (see Table 3), indicating that these variables did not exhibit the predictive power observed for tumour microstructure.

**Table 3:** Pearson correlation coefficients (r in the table) and their associated p-values (p in the table) between participants’ characteristics (years of education, age, pre- and post-surgical tumour volume, tumour grade, pre-surgery hippocampal volume and pre-surgery hippocampal activity) and mean overall cognitive decline values.

		Mean Cognition
Years of Education	*r*	−0.2249
	*p*	0.4600
Age	*r*	0.3927
	*p*	0.1845
Tumour_Vol_Pre	*r*	−0.1821
	*p*	0.5516
Tumour_Vol_Post	*r*	−0.0527
	*p*	0.8642
Tumour grade	*r*	0.2842
	*p*	0.3466
Hippocampus_Vol_Pre	*r*	0.2441
	*p*	0.4215
Activity_Hippoc_Pre	*r*	0.2637
	*p*	0.3840

#### Spatial similarity between P-Q and NODDI tumoural subregions

The spatial similarity between p-q- and NODDI-derived subregions that had shown significant associations with cognitive change was tested (Table 4). For this purpose: (1) NODDI-derived subregions were spatially compared to the group III subregion from the p-q analysis. The subregions that had the greatest spatial similarity with group III were C4 and C1 (based on DICE and percent occupancy); (2) p-q subregions were spatially compared to subregion C4. The subregions that showed the greatest spatial similarity with C4 were group IV and group III.

**Table 4:** (Upper rows) Sørensen-Dice similarity coefficient and percentage occupancy (% Occ) of each NODDI-derived subregion in the group III subregion. (Lower rows) Sørensen-Dice similarity coefficient and percentage occupancy (% Occ) of each p-q-derived subregion with the NODDI C4 subregion. Mean values and standard deviation (in parenthesis) across the sample.

Vox Gr III–C1		Vox Gr III–C2		Vox Gr III–C3		Vox Gr III–C4		Vox Gr III–C5	
DICE	% Occ	DICE	% Occ	DICE	% Occ	DICE	% Occ	DICE	% Occ
0.38 (0.12)	37.1 (10.4)	0.03 (0.03)	4.3 (4.9)	0.23 (0.17)	33.5 (20.4)	0.49 (0.18)	40.7 (17.8)	0.34 (0.19)	27.3 (13.4)

C4–Vox Gr I		C4–Vox Gr II		C4–Vox Gr III		C4–Vox Gr IV			
DICE	% Occ	DICE	% Occ	DICE	% Occ	DICE	% Occ		
0.00 (0.00)	4.0 (5.7)	0.00 (0.00)	1.3 (2.4)	0.49 (0.18)	65.6 (14.4)	0.64 (0.13)	71.0 (8.6)		

### Time-series analyses of BOLD signal

**Figure 5:**
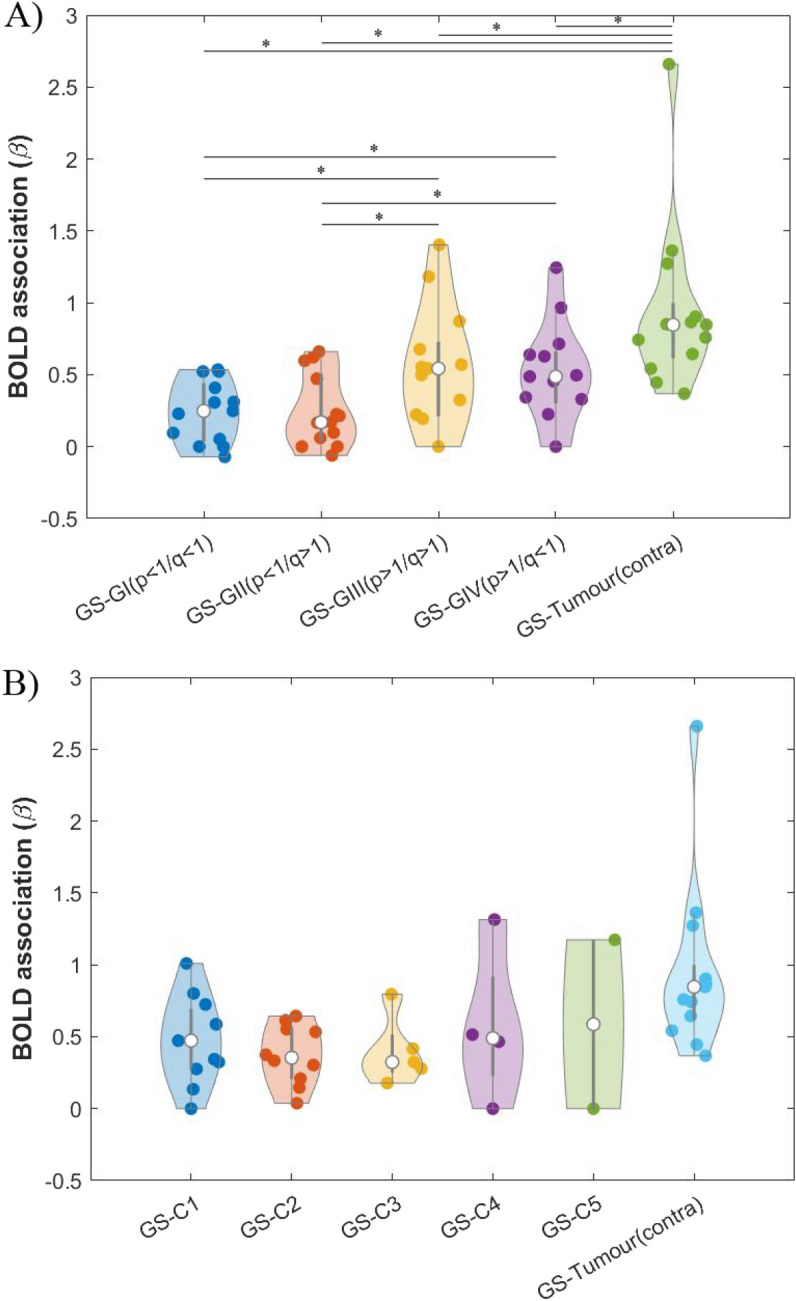
Distribution of β association values across participants (represented by individual dots) between: 1) BOLD signal derived from grey matter outside the tumour (GS), and 2) BOLD signal derived from the tumour subregions and from the region contralateral to the tumour (tumour (contra)). In the latter case, β values were calculated using a GS estimation that excluded the corresponding voxels contralateral to the tumour (to avoid overlapping between the independent and dependent variables). White dots represent the median of the data in each case; the thick grey line indicates the interquartile range. (**A**) DTI approach with tumour subregions: groups I, II, III and IV. * indicates significant differences, p-FDR < 0.01. (**B**) NODDI approach with tumour subregions: C1, C2, C3, C4 and C5.

The degree of functional coupling between p-q tumour subregions and GS, and between the contralateral region to the tumour and GS is represented in Fig. 5A. Statistical analyses revealed that the region contralateral to the tumour had a higher functional coupling with the GS in comparison with the tumour subregions (Friedman test: χ^2^ (4) = 39.433, p < 0.001, post hoc comparisons based on non-parametric Wilcoxon signed-rank test after FDR correction, p_cont-GI,FDR_ = 0.0033, p_cont-GII,FDR_ = 0.0033, p_cont-GIII,FDR_ = 0.0050, p_cont-GIV,FDR_ = 0.0033). Among the p-q tumour subregions, BOLD signals derived from group III and group IV subregions had significantly higher β values with the GS than those from group I and group II (p_GI-GIII,FDR_ = 0.0063, p_GI-GIV,FDR_ = 0.0063, p_GII-GIII,FDR_ = 0.0063, p_GII-GIV,FDR_ = 0.0063). The remainder of the comparisons were non-significant (p_GI-GII,FDR_ = 0.530, p_GIII-GIV,FDR_ = 0.2756).

The degree of functional coupling between NODDI-derived subregions and GS and between the contralateral region to the tumour and GS is represented in Fig. 5B. The contralateral region to the tumour showed a higher functional coupling with GS. Among the subregions, C5 had the greatest functional coupling with GS (Median = 0.587), followed by C4 and C1 subregions (Median = 0.490 and Median = 0.473 respectively). No statistical comparison of functional coupling was performed across the subregions due to the low number of tumours with subregions C3 (n = 5), C4 (n = 4) and C5 (n = 2).

**Figure 6:**
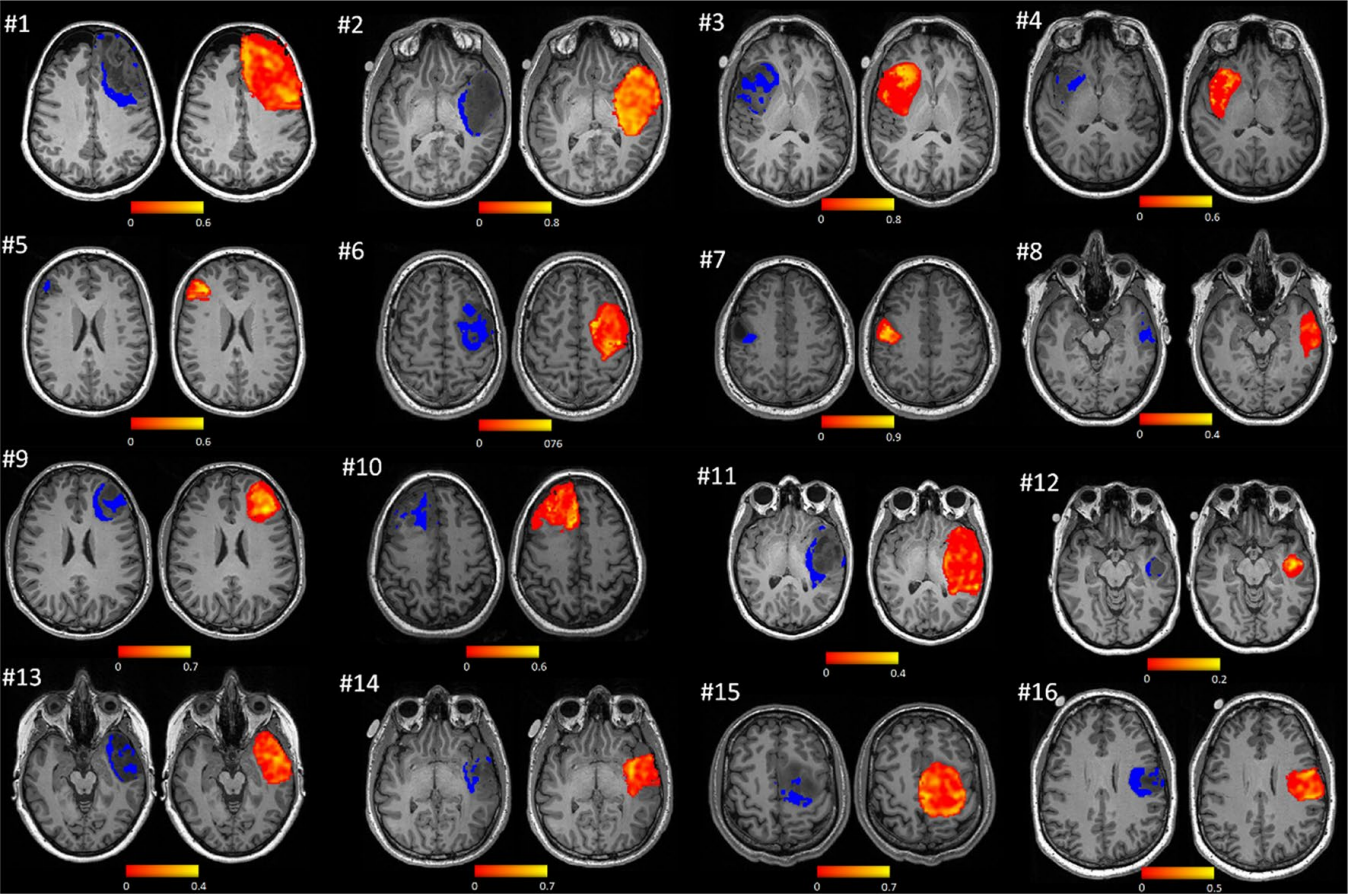
Distribution of group III subregions (left brains) and per-voxel functional coupling with GS (right brains). Participant numbers in the figure match those presented in Table 1.

Overall, these results reveal that group III subregions showed an association with cognitive decline and a correlation with GS. The spatial representation of these subregions (Fig. 6, left brains) suggests that they are primarily located in tumour margins where the voxel-wise coupling with GS was high (Fig. 6, right brains).

## Discussion

The present study combines structural and functional imaging of tumours in the pre-operative state and hypothesizes that microstructure impacts cognitive recovery after surgery by the functional embedding of tumours within brain functional networks. To test this hypothesis, we first characterized tumour microstructure by dividing the tumour into different subregions using two different methodological approaches. Once the tumour subregions were identified in the pre-operative state, we evaluated which had a greater impact on participants’ cognitive recovery after surgery. Finally, we examined the role of tumour microstructure in the functional interaction between the tumour and the unaffected brain in the pre-operative state. Our results support the notion that tumour subregions demonstrating the highest functional coupling with the GS play a crucial role in cognitive recovery after surgery.

Specifically, higher proportions of group III (*p* > 1/q > 1) within the tumour were associated with worse cognitive outcome after surgical intervention. The extent of group III subregions predicted mean overall cognitive change as well as deficits in verbal skills after surgery. It is worth noting that one of the inclusion criteria in the study was that the “tumour was located in or near speech-eloquent areas of the brain, i.e., regions that might be critical for speech comprehension and articulation”. Therefore, in terms of clinical treatment, the extent of the group III subregion is potentially capable of predicting recovery in this cognitive domain (i.e., verbal skills) that was compromised to some extent in all the participants. In addition, sensitivity analyses showed that other potential predictors of cognitive decline, such as years of education, age, tumour volume (pre- and post-surgery), tumour grade, pre-operative hippocampal activity and pre-operative hippocampal volume were not significantly associated with post-operative deficits in mean overall cognition.

Similar to that reported for glioblastomas^4^, we found that joint histogram analysis using DTI-p and -q values provided meaningful insights into tumour microstructure in both low- and high-grade gliomas. Following the nomenclature of the original work, the group III subregion was defined as the region that presented increased DTI-p and increased DTI-q values when compared to mean values of contralateral healthy tissue. Increased DTI-p values are thought to reflect lower cell density (i.e., more intercellular space), resulting in more isotropic diffusion. Alternatively, increased DTI-q reflects high anisotropy that might be due to the presence of intact fibres that facilitate tumour migration. An alternative explanation would be that an increased q represents compressed white matter tracts increasing anisotropic diffusion by reducing water diffusion perpendicular to the fibres. As a result of the increased fibre density, these white matter regions are particularly at risk of damage from surgery^4^.

We also found that group III was one of the tumour subregions showing the greatest functional coupling with the GS, indicative of an increased interaction between this tumour subregion and the unaffected brain. This suggests that higher proportions of group III within the tumour have a greater negative impact on participants’ cognitive recovery after surgery as tumour resection may remove a larger proportion of viable tissue -in terms of functional coupling- from the tumour. In addition, it is worth noting that the group III subregion was primarily located within the margins of the tumour in all participants (see Fig. 6, the group III partition displayed in dark blue). Altogether, decline in verbal skills might be attributed to surgical resection removing functional neurites in the tumour periphery.

Our results align with our prior research in overlapping cohorts^15,24^, indicating that participants’ cognitive decline after surgery was associated with a greater reduction (pre- vs post-surgery) in functional coupling between the BOLD signal within the tumour cavity and the GS^15,24^. Expanding on these findings, we initially identified that tumour tissue with distinct microstructural features exhibits varied GS coupling in the pre-operative state. Furthermore, our investigation revealed a specific tumour tissue, group III, that is uniquely associated with cognitive decline. This sheds new light on the challenges of understanding and treating eloquent areas of tumours, offering insights on how tumours interact with the neuronal microenvironment, a research question with major clinical relevance^30^. The group III partition may be either more eloquent itself or have a role in the plasticity of the functional network in which it is embedded.

The data-driven approach using measures derived from NODDI images showed that the presence of the C4 subregion was significantly associated with verbal skills decline after surgery. In this case, the C4 partition presents with low neurite density and high orientation coherence of neurites, resembling the characteristics of the group III subregion. However, the two subregions showed dissimilarities in their extension within the tumour: the mean percentage occupancy of the C4 partition (when present) was 74.6% (SD = 7.2) whereas the mean percentage occupancy of group III was 27.9% (SD = 11.5). These differences were also reflected in the Dice similarity index that had a mean of 0.49 (SD = 0.18) across participants. Interestingly, the effect sizes in the prediction of participants’ cognitive recovery in verbal skills were similar for both methodological approaches (P-Q: η^2^ = 0.619; NODDI: η^2^ = 0.529). Therefore, the two approaches lead to the general conclusion that tumour microstructure has an impact on cognitive recovery after surgery.

Summarizing, our results indicate that tumour microstructure is associated with cognitive recovery after surgery as well as with the extent of functional coupling with the unaffected brain. This work extends our understanding of the factors that determine cognitive outcomes for patients with brain tumours that could positively impact onco-functional balance when considering treatment options.

### Limitations

Accurately estimating the sample size for a study marked by heterogeneous conditions and complex interventions poses a significant challenge. The absence of prior (NODDI) MRI studies utilizing a similar design hinders the estimation of effect sizes required for a precise sample size calculation. Contrary to conventional methodologies, our investigation is structured to study the association between pre-surgical brain alterations, identified through MRI, and the cognitive changes induced by surgery. We anticipated that our patient-focused interventional approach would yield substantial effect sizes, enabling the detection of significant associations even within our limited sample size and perhaps even on an individual basis. We found that indeed some of the brain-cognition associations were of considerable strength (F-value > 7).

It is also worth noting that we are unable to provide histological validation as a ground truth of tumour microstructure. The differences found between both approaches may be due to methodological artifacts (e.g. MRI sequence, modelling error, definition or number of partitions) or to the fact that boundaries between tumour subregions are not distinct. In addition, the tumour subregions found in this work may represent either different tissue types or different progression stages of the subregions towards higher grades. On the other hand, we anticipated that different tumour subregions would show different coupling with the GS. In the case of necrotic tissue with no vascular circulation, we expected a reduced BOLD signal dominated by noise, which would lead to low functional coupling with the GS. However, this could not be formally assessed due to the lack of histological data. Future studies with histological validation and larger samples of patients should help clarify this issue.

It should also be noted that the post-operative period (2–5 weeks), where most of the participants underwent their neuropsychological assessment, might comprise transient surgical effects. Therefore, the present results should be validated over an extended period of cognitive follow-up.

Regarding the NODDI methodological approach, the implemented matching procedure set a threshold of Eucledian distances between centroids below 0.3 for matching tumour subregions across participants. This threshold was chosen as a trade-off between the number of distinct tumour subregions and their spatial overlap. However, the effect of changing this threshold should also be studied in future work.

### Supplementary Information

Supplementary Information.

## Data Availability

The datasets analysed during the current study available from the corresponding author on reasonable request.
