# Supplementary material for: Brain tumour microstructure is associated with post-surgical cognition
Source: Sci Rep. 2024 Mar 7;14:5646. doi: 10.1038/s41598-024-55130-5 (PMC10920778; doi:10.1038/s41598-024-55130-5)
Supplement: Supplementary file 1 — Supplementary Information. [file 41598_2024_55130_MOESM1_ESM.docx]

Brain tumour microstructure is associated with post-surgical cognition

**SUPPLEMENTARY INFORMATION**

**Neuropsychological assessment**

The neuropsychological assessment included 26 independent measures of cognitive function using previously validated tests ^1–3^. Specifically, the assessment included elements of both Weschler Adult Intelligence Scale IV tests, which have formerly been used to evaluate cognitive functioning in glioma patients ^4^, and the Brain Injury Rehabilitation Trust Memory and Information Processing Battery, which was specifically designed for patients with neurological injuries ^5^. Item-level details are shown in Table S1.

**Lesion masking and image co-registration**

Initially, an experienced neurosurgeon (MGH) experienced with MRI depicting brain tumours manually delineated the tumour on the pre-operative T1-weighted image slices for each patient. Each mask underwent additional refinement through ~~using~~ the Unified Segmentation with Lesion toolbox (https://github.com/CyclotronResearchCentre/USwithLesion, accessed on 31 April 2020) to enhance precision and reduce reliance on raters. Thus, masks included not only contrast-enhanced regions, but all abnormal regions (except for oedema, which was a contraindication to enrolment). This approach allowed for the compartmentalization of both the tumour and the surrounding tumour tissue based on their distinctive diffusion features.

Anatomic T1-weighted images were linearly co-registered to the DTI using advanced normalization tools (ANTs; <http://stnava.github.io/ANTs/>). Tumour masks were then transformed into the DTI space using the derived mapping. A tumour mask contralateral to the tumour was also generated for each patient. This mask was obtained by transforming the DTI space tumour mask into MNI space and then inverting the transverse spatial (‘X’) coordinates.

**Time-series analyses**

Framewise displacement (FD) was computed for each point in the time-series. To mitigate the potentially confounding effects of head motion, frames with FD > 0.4 were identified as outliers. The frame before and the two frames after the outliers were also considered outliers due to the delayed effect of motion in the BOLD signal. Frames labelled as outliers were removed from the time series. One scan with more than 50% of outliers was completely removed due to a poor signal-to-noise ratio (SNR) (n=3, see table S2) ^6^.

**References**

1. Vlaar AMM, Wade DT. The Adult Memory and Information Processing Battery (AMIPB) test of information-processing speed: a study of its reliability and feasibility in patients with multiple sclerosis. Clin Rehabil. 2003;17(4):386-393. doi:10.1191/0269215503cr625oa

2. Quental NBM, Brucki SMD, Bueno OFA. Visuospatial Function in Early Alzheimer’s Disease—The Use of the Visual Object and Space Perception (VOSP) Battery. PLoS One. 2013;8(7):e68398. doi:10.1371/journal.pone.0068398

3. Meyers JE, Zellinger MM, Kockler T, Wagner M, Miller RM. A Validated Seven-Subtest Short Form for the WAIS-IV. Appl Neuropsychol Adult. 2013;20(4):249-256. doi:10.1080/09084282.2012.710180

4. Zhou X, Grégoire J, Zhu J. The Flynn Effect and the Wechsler Scales. In: WAIS-IV Clinical Use and Interpretation. Elsevier; 2010:141-166. doi:10.1016/B978-0-12-375035-8.10005-9

5. Crawford JR, Garthwaite PH. Using regression equations built from summary data in the neuropsychological assessment of the individual case. Neuropsychology. 2007;21(5):611-620. doi:10.1037/0894-4105.21.5.611

6. Romero-Garcia R, Hart MG, Bethlehem RAI, et al. BOLD Coupling between Lesioned and Healthy Brain Is Associated with Glioma Patients’ Recovery. Cancers (Basel). 2021;13(19):5008. doi:10.3390/cancers13195008

| **Domain** | **Item name** |
| --- | --- |
| Attention | WAIS-IV.Digit.Span.Forward |
|  | WAIS-IV.Digit.Span.Backward |
|  | WAIS-IV.Digit.Symbol |
| Non-Verbal Skills | BMIPB.Complex.Figure.copy |
|  | VOSP.Object.Decision |
|  | VOSP.Number.Location |
|  | VOSP.Cube.Analysis |
| Memory | AMIPB.Story-Immediate.Recall |
|  | AMIPB.Story-Delayed.Recall |
|  | BMIPB.Word.List.A1-A15 |
|  | BMIPB.Word.List.A6 |
|  | BMIPB.Word.List  Word.Recognition |
|  | BMIPB.Word.List  List.Recognition |
|  | BMIPB.Complex.Figure  Immediate.Recall |
|  | BMIPB.Complex.Figure  Delayed.Recall. |
|  | BMIPB.Design.Learning-A1-A15 |
|  | BMIPB.Design.Learning-A6 |
|  | BMIPB.Design.Learning  Recognition |
|  | BMIPB.Design.Learning  Identification |
| Language | Letter.Fluency |
|  | Semantic.Fluency |
|  | Graded.Naming.Test |
|  | Syntactic.Speech.Comprehension |
| Executive  Functioning | Hayling.Initiation.(time) |
|  | Hayling.Inhibition.(time) |
|  | Hayling.Inhibition.(score) |
|  | Brixton |

**Table S1.** Items and domains used for neuropsychological assessment

|  | **MRI Scanning** | **Neuropsychological assessment** | | |
| --- | --- | --- | --- | --- |
| **#** | **Pre-operative** | **Pre-operative** | **Post-operative** | **Post-operative assessment period (number of days after surgery)** |
| **1** | Completed | Completed | Completed | 193 |
| **2** | Completed | Completed | Not completed | - |
| **3** | Completed | Completed | Completed | 18 |
| **4** | Completed  BOLD: Poor signal-to-noise ratio | Completed | Not completed | - |
| **5** | Completed | Completed | Completed | 18 |
| **6** | Completed | Completed | Completed | 10 |
| **7** | Completed | Completed | Completed | 24 |
| **8** | Completed  BOLD: Poor signal-to-noise ratio | Completed | Not completed | - |
| **9** | Completed | Completed | Completed | 164 |
| **10** | Completed | Completed | Completed | 18 |
| **11** | Completed | Completed | Completed | 18 |
| **12** | Completed | Completed | Completed | 19 |
| **13** | Completed  BOLD: Poor signal-to-noise ratio | Completed | Completed | 16 |
| **14** | Completed | Completed | Completed | 14 |
| **15** | Completed | Completed | Completed | 25 |
| **16** | Completed | Completed | Completed | 14 |

**Table S2.** MRI scans and neuropsychological assessments completed by each participant. # Participant identification number.


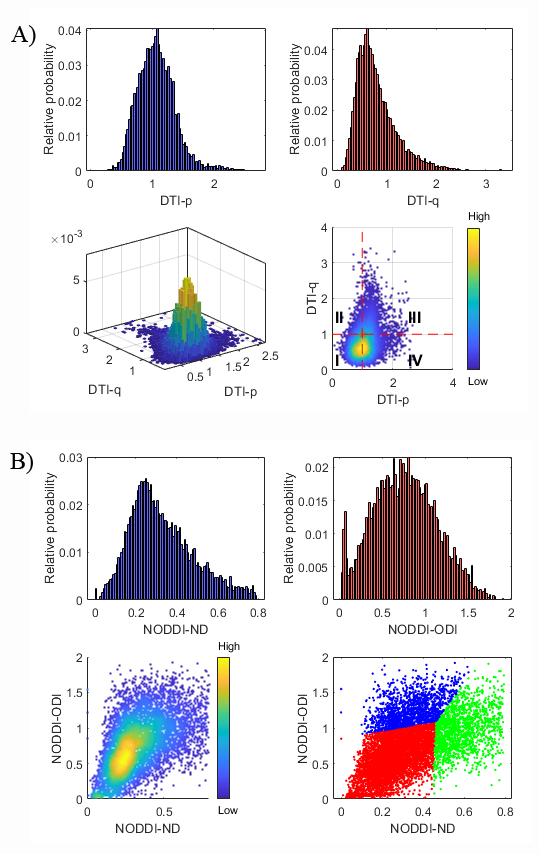


**Figure S1.** Illustration of the univariate and joint histogram analyses for both DTI (A) and NODDI (B) approaches performed on the same subject. **A) DTI** **(Upper row)** Univariate histograms of DTI-p and DTI-q maps (100 bins). **(Lower row)** The joint histogram was constructed with x- and y-axis representing DTI-p and -q values using 50 × 50 bins. **(Left)** The bin height of the joint histogram represented the relative probability of voxels falling into a specific DTI-p and -q range. **(Right)** Four voxel groups of DTI-p and -q abnormalities were obtained: I. Voxel group I (decreased DTI-p/decreased DTI-q); II. Voxel group II (decreased DTI-p/increased DTI-q); III. Voxel group III (increased DTI-p/increased DTI-q); IV. Voxel group IV (increased DTI-p/decreased DTI-q). **B) NODDI (Upper row)** Univariate histograms of NODDI-neurite density (ND) and NODDI-orientation dispersion index (ODI) maps. **(Lower row) (Left)** 2D joint histogram distribution. **(Right)** Three voxel groups defined after gaussian mixture model segmentation of the 2D joint histogram.
